# Case Report: Rapid progression to end-stage renal disease within 3 years in tuberous sclerosis complex challenging conventional risk stratification

**DOI:** 10.3389/fmed.2026.1873123

**Published:** 2026-06-24

**Authors:** Rushuang Yang, Xing Zheng, Ting Wang, Yanling Zhang

**Affiliations:** Department of Nephrology, The Third Hospital of Mianyang, Sichuan Mental Health Center, Mianyang, Sichuan, China

**Keywords:** angiomyolipoma, case report, end-stage renal disease, multidisciplinary team, tuberous sclerosis complex

## Abstract

Tuberous sclerosis complex (TSC) is a rare autosomal dominant disorder, with renal involvement typically presenting as angiomyolipomas (AML) and cysts. The progression to end-stage renal disease (ESRD) occurs in about 3.1% of cases. Current guidelines use AML size (>3 cm) as the threshold for initiating mTOR inhibitors, with less focus on rapidly progressing disease. This report describes a 28-year-old female with TSC whose renal function rapidly progressed from advanced CKD (stage G4A3) to anuria and ESRD within 3 years. She presented with significant proteinuria (3+, 5.94 g/24 h) and hematuria (3+) during pregnancy, but imaging showed no large tumors. Due to financial constraints, treatment was interrupted. Over approximately 30 months, her bilateral kidneys were replaced by diffuse AML, resulting in renal failure. This case challenges the traditional view of slow kidney progression in TSC and underscores the importance of early detection of proteinuria and hematuria as key warning signs. We recommend a multidisciplinary team (MDT) approach for high-risk patients, such as women of childbearing age, with dynamic monitoring of renal function and early proactive treatment to delay ESRD progression.

## Introduction

Tuberous sclerosis complex (TSC) is an autosomal dominant genetic disorder characterized by multisystem hamartomas, with renal involvement most commonly presenting as angiomyolipomas (AML) and cysts (1, 2). Although renal lesions are common in TSC patients, affecting approximately 57.5% of individuals, research on renal failure, proteinuria, and hematuria remains limited (3). Current clinical guidelines recommend the use of mTOR inhibitors for AMLs larger than 3 cm in diameter (4), but the mechanisms behind rapid renal decline in patients without significant AMLs have not been fully explored.

While the incidence of chronic kidney disease (CKD) in TSC patients is high, progression to end-stage renal disease (ESRD) is relatively rare, occurring in only 1 to 3.1% of cases (4, 5). However, in certain populations, renal function may deteriorate rapidly over a short period, suggesting that kidney damage may not solely result from direct compression or hemorrhage from AMLs but also from other mechanisms such as tubular hyperfiltration injury (6). Studies indicate that proteinuria, hematuria, and hyperfiltration are early warning signs of renal decline, particularly when AMLs have not reached intervention thresholds. In these cases, proteinuria often serves as a key indicator of kidney damage (7, 8).

Gender differences play an important role in TSC patients. Research has shown that female TSC patients have a higher incidence of AMLs and a higher rate of complications compared to males (9). The EXIST-2 study demonstrated that, due to the influence of sex hormones, AMLs in females may grow more rapidly, increasing the risk of renal complications. These findings suggest that females, especially those of childbearing age, face a higher risk in the renal progression of TSC, warranting special attention.

This report presents a 28-year-old female patient with TSC, whose renal function rapidly progressed from advanced CKD (stage G4A3) to anuria and ESRD within 3 years. Despite significant proteinuria and hematuria during pregnancy, the absence of large tumors on imaging and subsequent loss to follow-up resulted in delayed intervention. This led to the complete replacement of both kidneys with diffuse AML tissue within approximately 30 months. This case highlights the limitations of tumor size monitoring and emphasizes the need to establish a dynamic monitoring and early treatment model based on a multidisciplinary team (MDT) approach for high-risk groups, in order to delay the progression to renal failure.

## Case reports

**Figure 1:**
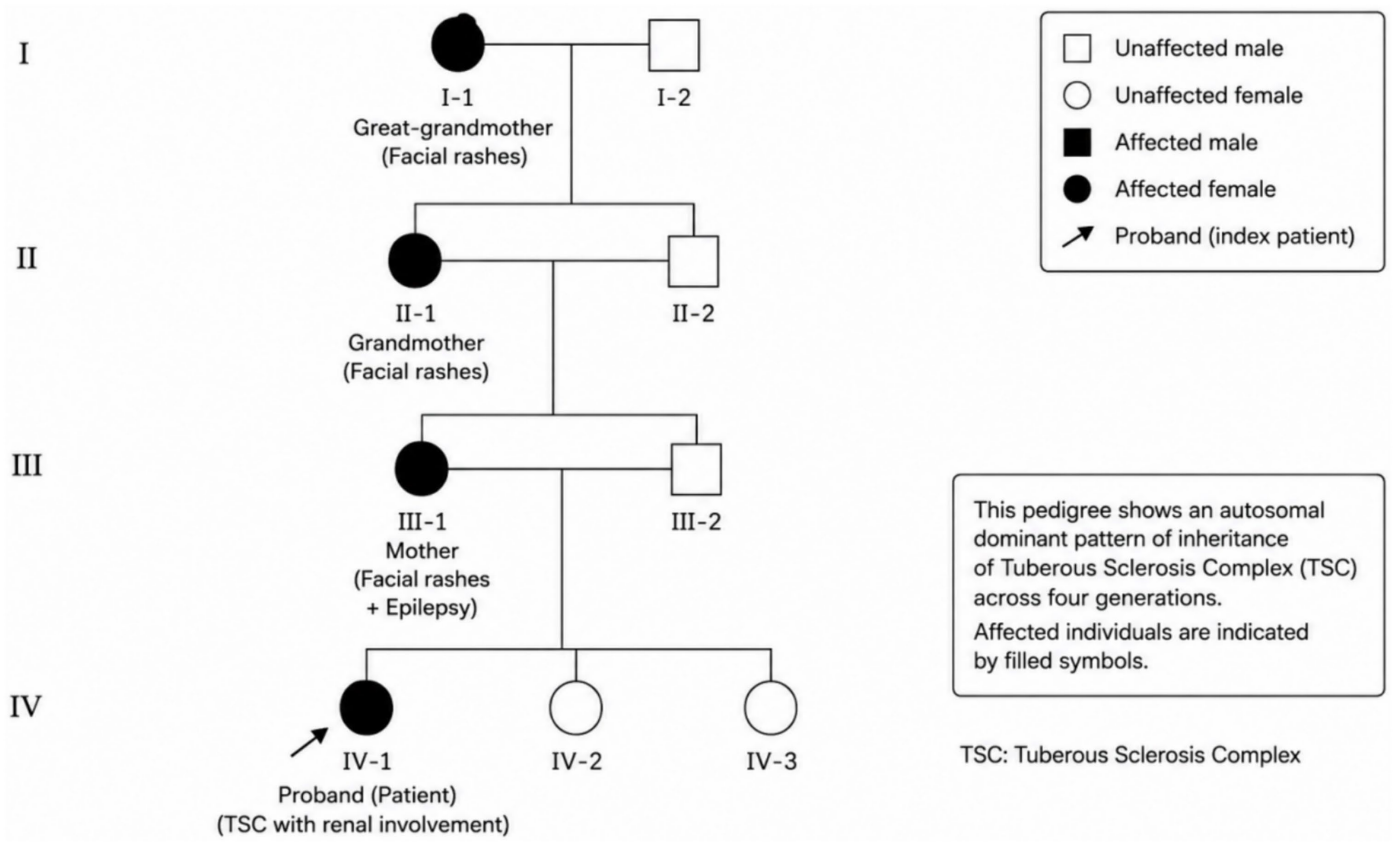
Pedigree of the family showing autosomal dominant inheritance of Tuberous Sclerosis Compler (TSC).

A 28-year-old female was admitted with anuria for 2 months, accompanied by nausea, vomiting, dyspnea, and severe fatigue. The patient had a significant family history of TSC on her maternal side; her mother, grandmother, and great-grandmother all had characteristic facial rashes, and her mother had a history of epilepsy. A pedigree chart illustrating the family history is shown in Figure 1.

**Figure 2:**
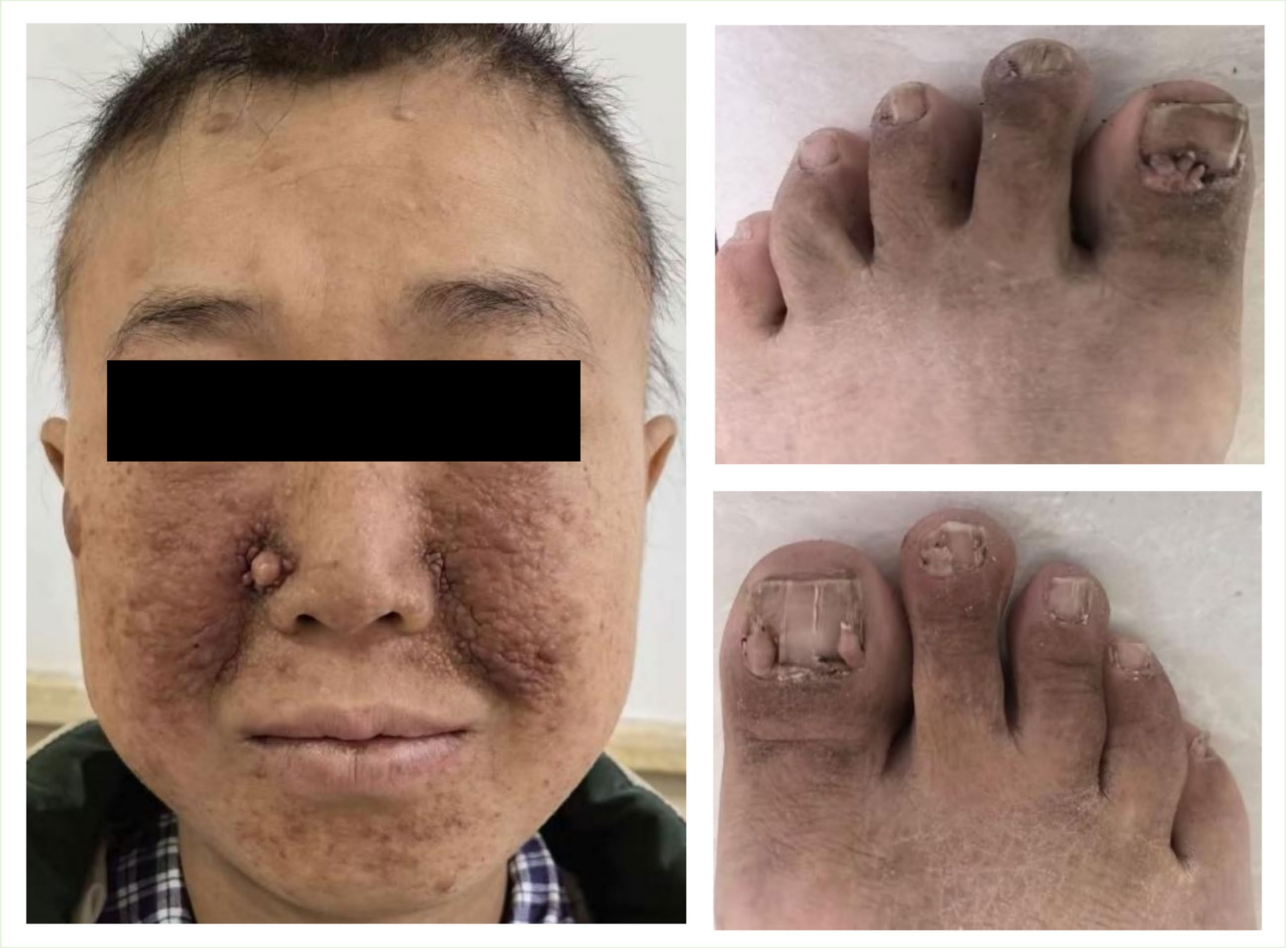
Dermatological findings of the patient. Facial features: Numerous (≥3) angiomyomas are seen on the cheeks and nasolabial folds, typically distributed over the zygomatic area. These angiomyomas appear as multiple red-brown, firm papules. Nail features: Multiple periungual and subungual fibromas (Cohen's tumors) (≥2) are present on the toenails, appearing as flesh-colored masses protruding from the nail beds.

Despite the genetic background, the patient was not diagnosed with TSC until her third pregnancy in June 2023, when she was 25 years old. At that time, she presented with hair loss and bilateral lower limb edema. Physical examination revealed typical TSC skin manifestations, including multiple yellowish-red angiofibromas on the cheeks (ranging in size from 2 to 15 mm) and several periungual fibromas on the toes (Figure 2), No clinical evidence of neurological involvement, including seizures, cognitive impairment, or developmental delay, was observed.

**Figure 3:**
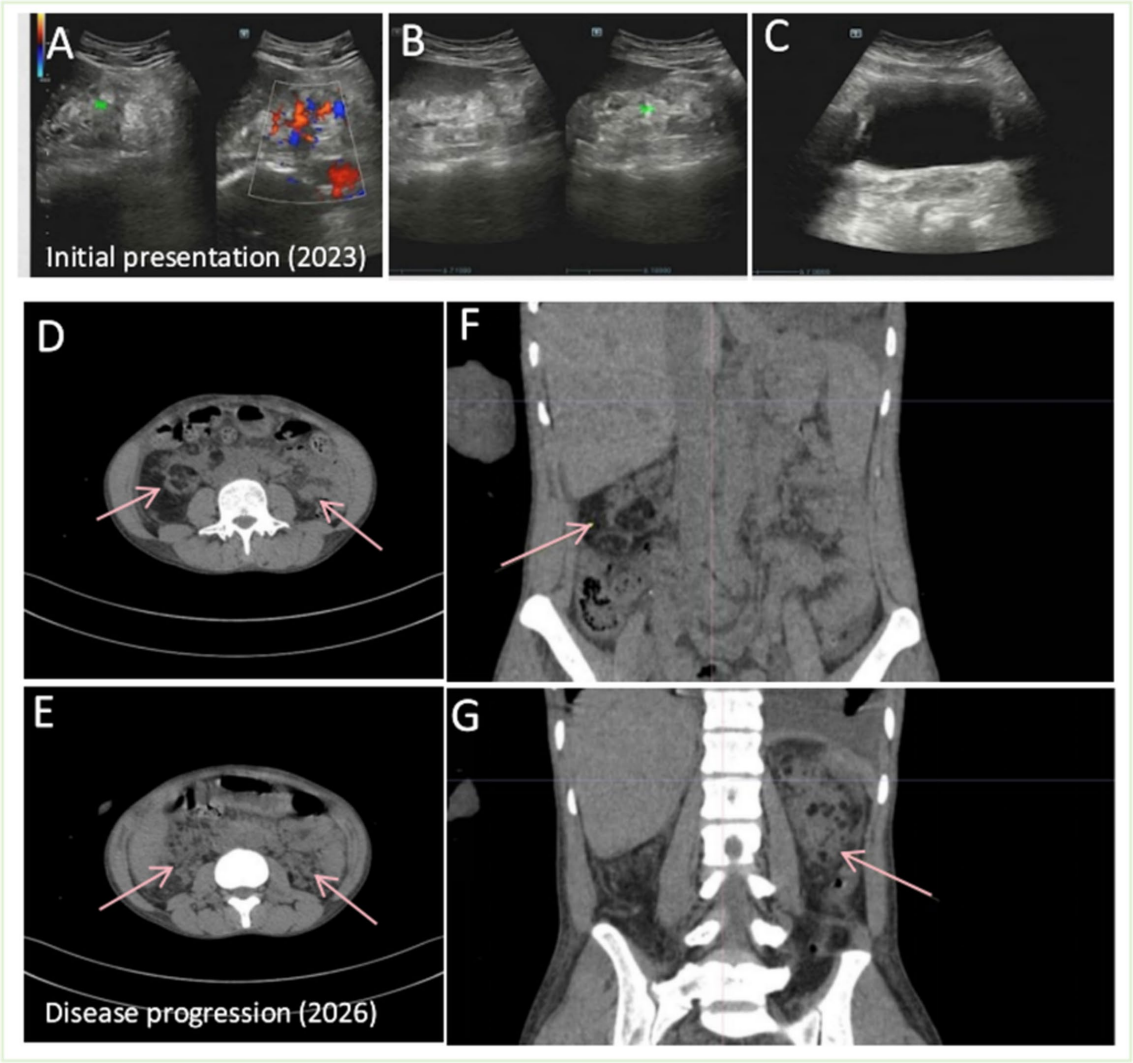
Evolution of renal imaging findings from 2023 to 2026. **(A–C)** Renal ultrasound obtained at initial presentation in 2023 demonstrating increased renal parenchymal echogenicity without evidence of significant angiomyolipomas or large space-occupying renal lesions. **(D,E)** Axial and **(F,G)** coronal abdominal CT images obtained in 2026 showing marked bilateral renal enlargement with numerous fat-density nodules consistent with diffuse angiomyolipomas. Mild perinephric fat stranding is present at the mid-to-lower poles. Arrows in **(F,G)** indicate the remaining renal parenchyma, suggesting extensive renal involvement.

Laboratory results in 2023 showed serum creatinine of 217 μmol/L, urea of 9 mmol/L, albumin of 30 g/L, and 24-h urine protein of 5.94 g, with urine occult blood 3 + and protein 3+. Lipid profile indicated cholesterol of 7.04 mmol/L and triglycerides of 3.95 mmol/L. Autoimmune screening (ANA, ANCA, and lupus anticoagulant) was negative, the detailed laboratory findings are summarized in Table 1. Despite a multidisciplinary team diagnosing TSC, imaging studies showed no large tumors meeting treatment criteria (Figure 3), and due to financial constraints, the patient did not receive mTOR inhibitor therapy or regular follow-up.

**Table 1:** Laboratory findings at initial presentation (2023) and ESRD stage (2026).

Parameter	2023	2026	Reference range
Serum creatinine	217	590	44–97 μmol/L
Urea	9	17.6	2.5–7.5 mmol/L
Albumin	30	39.7	40–55 g/L
24-h urine protein	5.94	—	<0.15 g/day
Urine protein	3+	3+	Negative
Hematuria (occult blood)	3+	±	Negative
Cholesterol	7.04	4.7	<5.2 mmol/L
Triglycerides	3.95	1.5	<1.7 mmol/L
LDL-C	4.0	3.1	<3.4 mmol/L
HbA1c	5.3%	5.1%	4.0–6.0%
ANA	Negative	Negative	Negative
ANCA	Negative	Negative	Negative
Lupus anticoagulant	Negative	Negative	Negative
Immunoglobulin profile	Normal	Normal	Normal
Serum protein electrophoresis	Negative	Negative	Negative
Immunofixation electrophoresis	Negative	Negative	Negative
Hemoglobin	124	59	120–160 g/L
iPTH	—	336	15–65 pg./mL
β2-microglobulin	—	45.9	0.8–2.2 mg/L

By January 2026, the patient had progressed to ESRD. Upon re-admission, clinical examination revealed anemia, persistent facial angiofibromas, and periungual fibromas. Laboratory tests showed serum creatinine had risen to 590 μmol/L, and hemoglobin had dropped sharply to 59 g/L. Urinalysis showed persistent proteinuria (3+).

Abdominal computed tomography (CT) revealed significant bilateral kidney enlargement, with the renal parenchyma almost completely replaced by numerous fat-dense nodules (Figure 3), characteristic of diffuse renal AML. According to the 2021 International TSC diagnostic criteria, the patient met three major criteria for TSC diagnosis: facial angiofibromas (≥3), ungual fibromas (≥2), and renal AMLs (≥2).

Given the patient’s anuria and progressive renal failure, she was initiated on maintenance hemodialysis via a tunneled cuffed internal jugular vein catheter (three times per week). Subsequently, under renal replacement therapy, the patient’s condition stabilized.

## Discussion

CKD in patients with TSC typically occurs in the presence of large, space-occupying lesions that invade normal renal parenchyma, often requiring surgical debulking or selective embolization to remove part of the renal parenchyma (10). However, this case challenges this traditional view, documenting a rapidly progressing natural course: a 28-year-old female whose renal function quickly progressed from advanced CKD (stage G4A3) to anuria and ultimately to ESRD within approximately 30 months.

Importantly, in this patient, significant proteinuria and hematuria were already present in 2023 despite the absence of large angiomyolipomas on imaging. These findings are not typical of classical angiomyolipoma-related renal involvement and may suggest an alternative pattern of renal injury. Therefore, alternative mechanisms of renal damage should be considered beyond conventional angiomyolipoma-driven parenchymal loss in TSC. By excluding common chronic kidney disease causes such as diabetes, systemic lupus erythematosus, and vasculitis, and considering that the patient had not been exposed to nephrotoxic drugs, the deterioration of her condition is most likely related to progressive TSC-associated renal involvement. By the time of admission in 2026, abdominal CT showed that the renal architecture was nearly entirely replaced by diffuse fat-density nodules (Figure 2), characteristic of diffuse renal AML. This suggests that the patient’s renal failure was not caused by the expansion of a single lesion but by the rapid growth and fusion of multiple small lesions. This diffuse growth pattern indicates that if clinical treatment relies solely on tumor size (e.g., >3 cm) as the intervention threshold, the treatment window for preserving renal units may be missed before the tumor reaches the “giant” size visible on imaging.

In addition, this case highlights the potential impact of pregnancy on the rapid decline of kidney function in Tuberous Sclerosis Complex patients. TSC-related angiomyolipomas are hormonally sensitive, and estrogen and progesterone receptor expression may contribute to accelerated lesion progression and renal functional decline during pregnancy. The proteinuria and hematuria observed during the patient’s pregnancy in 2023 were not only markers of renal injury but also potential early indicators of more diffuse renal involvement. At the same time, standard supportive therapy for chronic kidney disease, including blood pressure control, renin–angiotensin system inhibition, and lipid-lowering therapy, was not continuously implemented due to pregnancy, socioeconomic constraints, and lack of regular follow-up, which may have been one of the contributing factors to the rapid progression of renal dysfunction.

Recent studies have expanded the understanding of renal involvement in Tuberous Sclerosis Complex beyond angiomyolipoma-driven parenchymal loss. Kronick et al. described a microscopic variant of TSC-associated kidney disease characterized by progressive kidney function decline despite the absence of large renal lesions (11), while Sakhi et al. further reported that chronic kidney disease may occur in TSC patients without large angiomyolipomas or prior renal interventions (12). Notably, Sakhi et al. also described stabilization of kidney function following everolimus treatment, suggesting a potential role for mTOR inhibition beyond the current indication of large AMLs. However, evidence remains limited and the long-term renal benefits of this approach remain uncertain.

Our patient shared several features with these recently described cases, including heavy proteinuria, hematuria, and rapid deterioration of kidney function despite the absence of significant AML burden at presentation. While renal biopsy was considered, it was not performed because the patient declined invasive diagnostic procedures during follow-up and was not suitable for biopsy during pregnancy. Therefore, although histopathological confirmation is lacking, a microscopic variant of TSC-associated kidney disease cannot be excluded.

This case underscores the unique nature of rapid renal deterioration in TSC patients, particularly in the absence of large AMLs. To prevent similar situations, the following key lessons may be worth considering: (1) Regular assessment of creatinine and urinary protein should be conducted, and treatment should be initiated early upon detection of proteinuria or hematuria, even if the AML lesion is less than 3 cm. (2) Even in the absence of large tumors on imaging, rapid renal decline may be caused by diffuse, small lesion-type TSC nephropathy, which warrants increased attention to such variants. (3) High-risk patients, particularly women of childbearing age, should undergo dynamic monitoring through multidisciplinary collaboration, allowing for early detection of rapid progression and timely intervention.

Although there is currently no unified guideline on how to manage AMLs smaller than 3 cm, this case suggests that tumor size is not always proportional to the severity of renal damage. Future research should further explore how functional assessments and early interventions can protect renal function when early renal damage becomes apparent.

This study has several limitations. First, genetic testing was not performed to confirm tuberous sclerosis complex. Second, histopathological confirmation of the renal lesions was not obtained, as the patient declined invasive diagnostic procedures.

## Data Availability

The original contributions presented in the study are included in the article/supplementary material, further inquiries can be directed to the corresponding author.
